# Computational Multiscale Toxicodynamic Modeling of Silver and Carbon Nanoparticle Effects on Mouse Lung Function

**DOI:** 10.1371/journal.pone.0080917

**Published:** 2013-12-03

**Authors:** Dwaipayan Mukherjee, Danielle Botelho, Andrew J. Gow, Junfeng Zhang, Panos G. Georgopoulos

**Affiliations:** 1 Department of Environmental and Occupational Medicine, Robert Wood Johnson Medical School, Rutgers University, Piscataway, New Jersey, United States of America; 2 Department of Chemical and Biochemical Engineering, Rutgers University, Piscataway, New Jersey, United States of America; 3 Department of Pharmacology and Toxicology, Rutgers University, Piscataway, New Jersey, United States of America; 4 Department of Preventive Medicine, Keck School of Medicine, University of Southern California, California, United States of America; Emory University School of Medicine, United States of America

## Abstract

A computational, multiscale toxicodynamic model has been developed to quantify and predict pulmonary effects due to uptake of engineered nanomaterials (ENMs) in mice. The model consists of a collection of coupled toxicodynamic modules, that were independently developed and tested using information obtained from the literature. The modules were developed to describe the dynamics of tissue with explicit focus on the cells and the surfactant chemicals that regulate the process of breathing, as well as the response of the pulmonary system to xenobiotics. Alveolar type I and type II cells, and alveolar macrophages were included in the model, along with surfactant phospholipids and surfactant proteins, to account for processes occurring at multiple biological scales, coupling cellular and surfactant dynamics affected by nanoparticle exposure, and linking the effects to tissue-level lung function changes. Nanoparticle properties such as size, surface chemistry, and zeta potential were explicitly considered in modeling the interactions of these particles with biological media. The model predictions were compared with *in vivo* lung function response measurements in mice and analysis of mice lung lavage fluid following exposures to silver and carbon nanoparticles. The predictions were found to follow the trends of observed changes in mouse surfactant composition over 7 days post dosing, and are in good agreement with the observed changes in mouse lung function over the same period of time.

## Introduction

Production of engineered nanomaterials (ENMs) is rapidly rising globally, as is their usage in consumer products, resulting in increased human contact on a daily basis. As inhalation is a major exposure route for many ENMs, understanding the interaction between ENMs and the components of the lung lining fluid and the cells of the alveolar region becomes essential in determining the tissue-specific and organism level effects of ENMs. ENMs have novel physical and chemical properties, stemming from their size (1–100 nm) and can undergo dynamic changes when interacting with biological systems [1], which would be intrinsically different from the effects seen with small molecules or with micron-sized particles. This produces a high degree of uncertainty associated with the toxic effects produced by engineered nanomaterials, and the mechanisms behind such effects remain to be fully characterized [2].

The work presented here is part of a larger international effort across multiple universities to develop a modular, multiscale, biologically-based system to provide a meaningful and generalizable risk assessment framework, by utilizing *in vitro* and *in vivo* measurements and also relevant mechanistic information available in the scientific literature. The system is meant to be implemented for specific ENMs to provide mechanistic descriptions for the toxicodynamic effects of ENMs at multiple biological scales. In the present work, a detailed, multiscale computational toxicodynamic model has been developed to analyze the effects of inhaled nanoparticles on the respiratory system of mice, using silver (nAg) and carbon black (CB) nanoparticles as examples.

The toxicodynamic model considers the effects of nanoparticle inhalation on biological and mechanical responses in the lung. The entire multiscale system has been decomposed into four functional modules to capture the molecular, cellular, and immune reactivities of ENMs with the biological components of the alveolar microenvironment. Module I considers the binding of surfactant to nanoparticles once they reach the alveolar surface, which results in surfactant depletion and affects lung function. Module II considers the balance of surfactant in the system considering surfactant secretion by cells, adsorption to the interface, and surfactant recycling. Module III considers particle uptake by type I and type II cells and macrophages lining the pulmonary alveolar wall. Module IV considers the inflammatory dynamics in the lung, involving cytokines, neutrophils and other immune cells. This article focuses on Modules I, II, & III only, because these are most relevant to the pulmonary endpoints being addressed here. The three modules have been linked to an organism level lung mechanics module which considers changes in pulmonary function due to the interactions of ENMs at the alveolar microenvironment. The model comprising the individual modules successfully captures the kinetics of surfactant phospholipid and proteins shown in BALF samples from exposed mice. The model also links alveolar surfactant amounts to overall lung function in mice using linear parameters quantifying surfactant-induced effects at different breathing conditions. This is the first attempt known to the authors to link physiological and biochemical effects occurring at multiple scales within the pulmonary system using a computational model.

## Methods

The protocol for the *in vivo* measurements in mice was approved by the Rutgers University Institutional Animal Care and Use Committee (IACUC - Protocol Number: 06–028). The study was conducted in accordance with the recommendations in the Guide for the Care and Use of Laboratory Animals of the National Institutes of Health.

### Modeling Surfactant Kinetics - Module II

**Figure 1:**
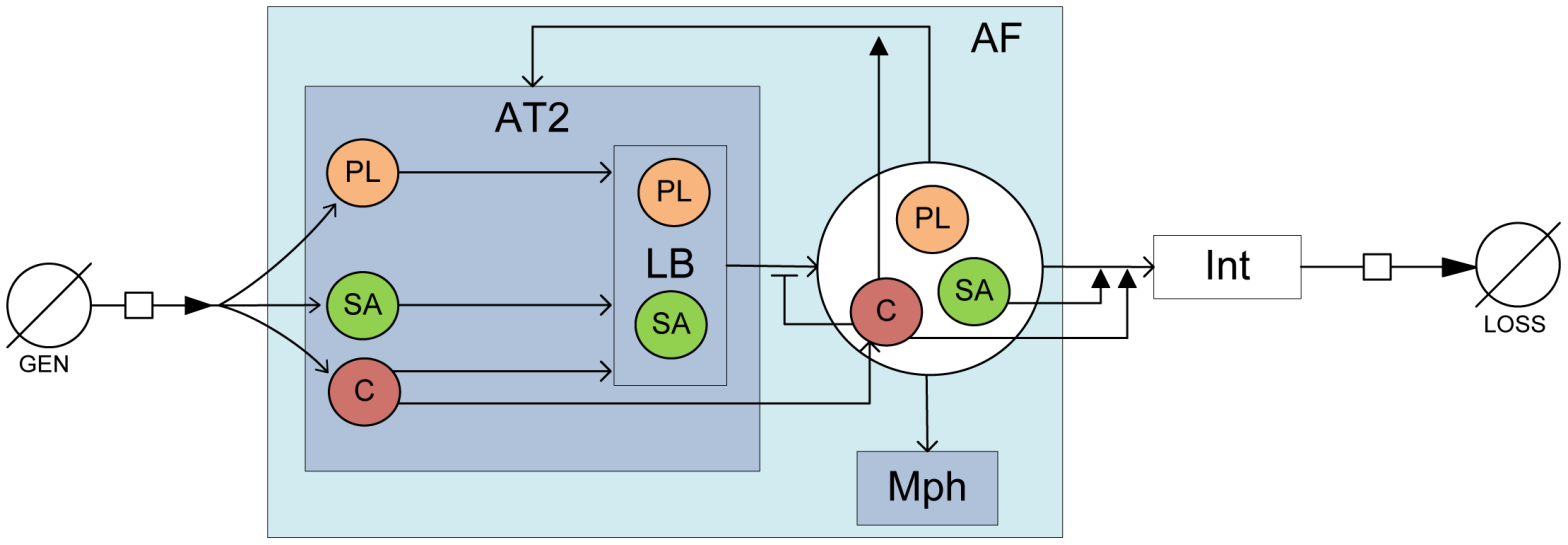
Detailed schematic of compartmental model for surfactant dynamics consisting of surfactant components Phospholipids (PL), Surface-active lipoprotiens (SA), and Collectins (C). Physiological compartments considered are alveolar type II cells (T2), lamellar bodies (LB) within type II cells, alveolar macrophages (Mph), alveolar fluid (AF), alveolar air-liquid interface(Int), surfactant generation in cells (GEN), and surfactant loss to airways (Loss).

**Figure 2:**
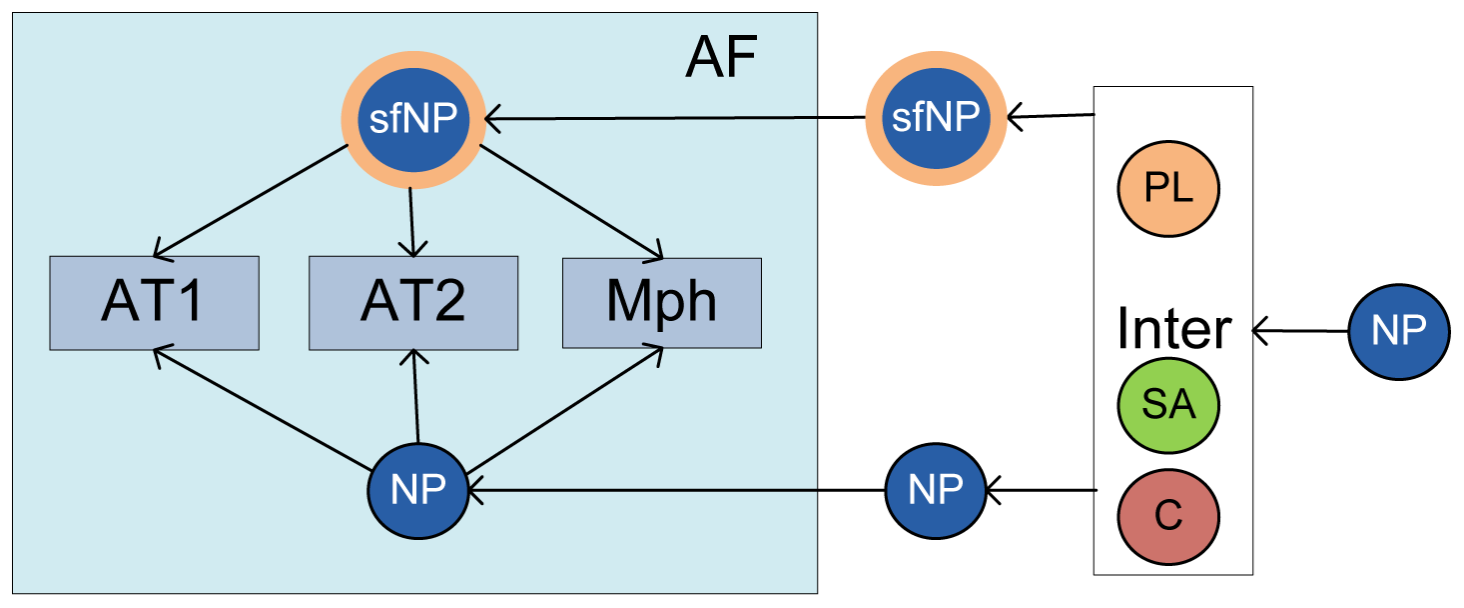
Detailed schematic of compartmental model for Modules I & III considering surfactant binding with NPs and NP uptake by alveolar Type I and Type II cells and macrophages.

The compartmental model developed here, along with the various tissue compartments and cells involved in the model are shown in Figures 1 and 2. Pulmonary surfactant is composed of 90% of phospholipids, and about 10% of lipoproteins [3]. The lipoproteins present in pulmonary surfactant are generally classified into 4 types: Surfactant proteins (SP) A, B, C, and D. SP-B and SP-C are lipophilic proteins which are involved in modulating the surface-active function of pulmonary surfactant. They have been represented in the model as surface-active proteins, SA. SP-A and SP-D are associated with immune response to xenobiotics and have been represented as collectins, C. The molecular weights of the various components of pulmonary surfactant are summarized in Table 1. The molecular weight of PL is calculated by averaging the molecular weights of its constituent species based on their percentage. The various steps involved in the processing of pulmonary surfactant [4] can be summarized as:

**Table 1:** Summary of Molecular weights of surfactant components.

Surfactant proteins	Mol.Wt(in kDa) [42]
SP-A	26
SP-B	8.7
SP-C	3.7
SP-D	39
**PL species**	**Mol.Wt(in kDa)** *
PC	760.076 (78)
PE	471.609 (3)
PS	547.17 (5)
PG	787.383 (7)
SM	646.505 (2)
CL	400.637 (5)
Mean for PL	722.445

Figures in brackets denote percentage composition in alveolar PL (data from [3]).

* Source: Avanti Polar Lipids, Co.

- Secretion of surfactant components PL, SA, & C (without intermediate role of lamellar bodies (LB)) [4], [5]
- Exocytosis of LB and release of surfactant into the alveolar fluid (AF)
- Processing of surfactant into tubular myelin
- Migration of tubular myelin from the AF to the alveolar air-liquid interface (Int) [6]
- Formation of the surfactant layer at the interface
- Collapse of surfactant layer at the interface
- Recycling of surfactant components into type II cells

#### Surfactant secretion

Surfactant components are secreted by alveolar type II cells (AT2) into lamellar bodies (LB) [7]. The process of secretion of the surfactant components is considered to occur in two steps - the secretion of the chemicals into LB and the exocytosis of LB into the AF. There is considerable debate in published literature on the exact secretory pathway of the surfactant proteins. The surface-active (SA) proteins, SP-B and SP-C, are most certainly secreted into the LB, but there is evidence that the collectins, SP-A and SP-D, can be secreted in an LB-independent manner [8]. Some researchers [9] have shown most SP-A to be secreted constitutively independent of LB. However, Fisher *et al.* [10] have shown that secretion of SP-A into LB precedes its secretion into the hypophase. But there is increasing evidence that both pathways are involved in the secretion of SP-A [11]. So, both pathways of secretion have been considered for collectins. The lamellar bodies are exocytosed from the type II cells and the secreted surfactant components released. The rate of LB exocytosis has been estimated from Martini *et al.* [1] to be 239 nmol/h/g lung. Surfactant components are also known to be recycled back into the type II cell [5], [12]. In addition to these processes of removal of surfactant components, macrophages ingest some surfactant and some amounts are lost via the airways [5]. Macrophage uptake of surfactant components has been studied by Gurel *et al.* [15] and the rate constants have been estimated from their work. Pettenazzo *et al.* [14] and others have found airway loss of surfactant to be limited to 3% over 24 hours. Accordingly, a fractional loss term is included for loss of surfactant to the airways.

#### Surfactant adsorption

The surfactant components in the hypo form concentrate film-like structures called tubular myelin [5] which are then adsorbed onto the alveolar air-liquid interface. These processes have been modeled as a single adsorption process, where the three surfactant components adsorb at different rates onto the alveolar surface lining. The rate of adsorption is influenced by surfactant concentration in the sub-phase (hypophase) and also by the concentration in the pre-existing alveolar surface film [15]. Adsorption rate of surfactant components, 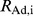 is estimated as: 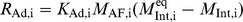, where, 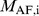 is the bulk amount of component 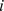 in the alveolar fluid, 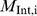 is the surface amount, and 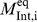 is the equilibrium surface amount which corresponds to the minimum surface tension reached at the end of expiration. The adsorption rate constant is estimated using data from Walters *et al.* [15]. Protein and lipid components of surfactant are separated during adsorption into the surface layer [5]. Since they are part of the same tubular myelin when they get adsorbed into the interface, the adsorption and depletion rates of SA and C are considered to be identical to the adsorption rate of PL but the equilibrium amounts, 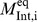 for each component would be different. The surface film also splits during the breathing cycle and the surfactant components are returned to the hypophase [16], which is modeled by a desorption term.

#### Regulation of surfactant dynamics

The various components of pulmonary surfactant do not function independently. The surfactant proteins are involved in various functions in the regulation of secretion, adsorption and recycling of phospholipids in the alveolar sub-phase [5]. SP-A binds strongly to PL and promotes the formation of the tubular myelin and the interfacial surfactant film [3], [17]. SP-B and SP-C promote adsorption of PL to the alveolar surface and aid in surface tension reduction by helping spread the lipids [18]. SP-A has also been found to inhibit the secretion of PL by Type II cells [12] and to promote the recycling of PL back into Type II cells (Chapters 4 and 7 in [19]). The regulation of these processes has been quantified using results from published studies.

#### Description of a five-compartment mathematical model

The mathematical model for Module II, considers the dynamics in the alveolar lining fluid and involves five compartments, namely type II cells (AT2), lamellar bodies (LB), alveolar fluid (AF), alveolar air-liquid interface (Int), and a purely mathematical compartment called ‘Loss’, which considers the net loss of surfactant from the system. The module with its constituent compartments is schematically depicted in Figure 1. The mass balance equations involving the five compartments are shown below. 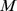 represents the amount of a particular species in mol, and 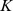 stands for the rate constant for a particular process in min^−1^. The subscript ‘I’ represents the three surfactant components considered - PL, SA, and C.
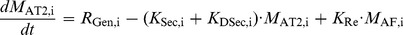
(1)

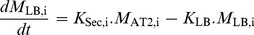
(2)

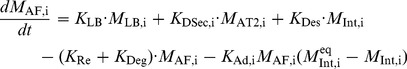
(3)

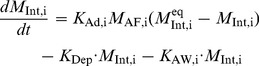
(4)

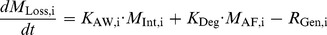
(5)


Here 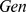 stands for generation, 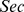 for secretion, 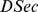 for direct secretion, 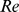 for recycle, 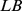 for exocytosis of lamellar bodies, 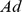 for adsorption, 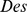 for desorption, 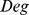 for degradation, and 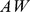 for airway loss. 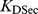 is the rate constant for direct constitutive secretion into the alveolar fluid, which is known to happen for collectins [11] and does not exist for PL or SA. So 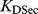 is zero for PL and SA. There are a total of 5×3 = 15 differential equations for the mass balance of surfactant components.

The regulatory kinetics of surfactant components on the various surfactant delivery processes as described above in *Regulation of surfactant dynamics* are mathematically described in the equations below. 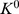 stands for the rate constant without the regulation effect, 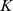 stands for the rate constant with regulation, and 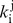 represents the regulatory rate constant of species 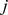 on process 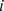. 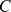 represents the concentration of the particular component in the alveolar fluid in mol/ml.
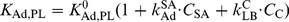
(6)

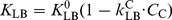
(7)

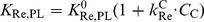
(8)


#### Parameter estimation

The estimation of the rate constants described in the above equations has been carried out using results from published *in vitro* and *in vivo* studies. Physiological parameters for mice were estimated from the literature and, wherever unavailable, were scaled from other species using body weight (Table 2). The rate constants whose values were explicitly estimated from the literature are summarized in Table 3. Only 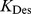, 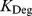, and 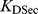 were not readily available in the literature and were estimated based on a steady-state analysis. The pulmonary surfactant sub-system maintains the levels of the various surfactant components steady at a physiological level to maintain the functions of surfactant in the lung. The steady-state values of various components in the physiological compartments are summarized in Table 4. The steady state in AT2 is maintained explicitly in the mathematical model by considering the generation term as: 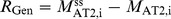. The lamellar body (LB) is a transient entity and is constantly forming and dividing and there cannot be any biological steady state for it. So the steady state analysis considers a steady state in the alveolar fluid (AF) and alveolar interface (Int). Accordingly based on Equations 3 and 4, the steady state for PL and SA (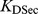 = 0) can be represented as:
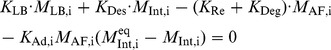
(9)

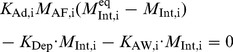
(10)


**Table 2:** Morphological parameters of the pulmonary system used in the model.

Parameter	Value	Reference
Total lung volume	1.43 ml	Measured in mice by [43]
Total lung mass	0.43 g	Measured in mice by [43]
Volume of alveolar fluid	10 ml/kg BW	Reported in sheep by [44]
Alveolar surface	0.0082 m^2^	For 0.3 ml lung [45]
Thickness of alveolar interface	0.2 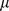 m	Reported in rats by [46]
Volume of single Type II cell	385.7 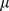 m^3^	Number weighted mean in rats [47]
No. of Type II cells	9.13×10^7^ per g lung	Reported in mice by [13]
No. of alveolar macrophages	1.66×10^5^	Reported in mice by [48]
Volume of LB per Type II cell	61.3 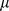 m^3^	Reported in rats by [47]
No. of LB per Type II cell	150	Estimated in rats by [49]

**Table 3:** Values of regulatory parameters.

Parameter	Description	Literature value	Reference
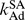	Parameter for activation of surfactant adsorption by SA	7.312×10^4^ per 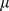 mol/ml of SA	[50]
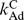	Parameter for activation of surfactant adsorption by C	2.185×10^5^ per 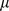 mol/ml of C	[50]
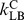	Parameter for inhibition of surfactant secretion by C	1.7342×10^3^ per 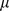 mol/ml of C	[51]
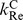	Parameter for activation of surfactant recycling by C	3.078×10^4^ per 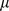 mol/ml of C	[52]

**Table 4:** Reference values of PL, SA, & C amounts in various compartments (in 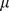
mol/g lung).

	Type IIcell	LamellarBody	Alveolarfluid†	Alveolarinterface
PL	10†	1.14‡	0.0818	1.46‡
SA	0.175††	1.995×10^−2^ ††	0.0035	0.0301◊
C	1.7848††	6.8×10^−6^ ††	0.0146	0.0151◊

† Original data from young humans, scaled by lung weight [53].

‡ Original data from pigs, scaled by lung weight [7].

†† Original data for LB from rats, AT2 composition calculated by assuming identical proportion as in LB [54].

◊Original data from bovine surfactant [55].

The two unknown parameters 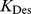, and 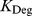 are estimated for PL and SA using the steady state equations 9 and 10. For C, the equation for alveolar fluid would be different because of the direct secretion pathway of C. Equation 9 would be modified as:
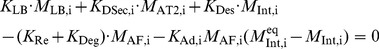
(11)



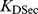
 is estimated from Equation 11 assuming the value of 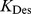 for C to be equal to that of PL and SA and 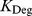 for C estimated from [13].

The parameter values estimated from the literature are based on *in vivo* studies and hence implicitly include the regulatory effects. However, since the effects of regulation are explicitly considered in the model, the process parameters 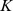 need to be considered separate from the regulatory parameters 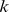. The parameter values are optimized using the *fmincon* subroutine in Matlab, using the literature values of 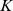 and 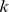 as initial estimates. The final optimized parameter values (along with the initial estimates) are summarized in Table 5.

**Table 5:** Parameter values after optimization (in min^−1^) (Values in brackets denote available initial estimates from the literature).

Parameter	PL	SA	C	Reference
Secretion, *K*_Sec_	3.833×10^−5^	3.833×10^−5^	3.833×10^−5^	(3.833×10^−5^)[7]
Direct secretion, *K*_CSec_	–	–	8.456×10^−5^	–
LB exocytosis, *K*_LB_	0.002	0.002	0.002	(0.0035) [7]
Recycle, *K*_Re_	1.798×10^−5^	1.798×10^−5^	9.63×10^−4^	(0.0025) [7]
Adsorption, *K*_Ad_	3.795×10^−5^	0.0402	0.0402	(0.0402) [15]
Desorption, *K*_Des_	0.0053	0.0053	0.0053	–
Degradation, *K*_Deg_	0.0466	0.0611	0.0014	–
Airway Loss, *K*_AW_	2.083×10^−5^	2.083×10^−5^	2.083×10^−5^	(2.083×10^−5^)[14]

Only parameters 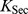, & 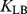, 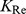, 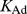, & 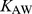 have initial estimates from the literature. The others are estimated based on steady-state analysis of the system.

### Nanoparticle Interaction with Surfactant - Module I

Nanoparticles, like other xenobiotics, are arrested by the interfacial surfactant layer after reaching the terminal airways and are coated by a layer of surfactant. Presence of particles on the alveolar surface might cause surfactant dysfunction by two mechanisms: direct and indirect [3]. Inhaled NPs bind to a fraction of the surfactant making it unavailable for adsorption and spreading on the alveolar interface and thus limiting the capacity of the alveolar to reduce surface tension [20]. Besides the direct interaction, presence of nanoparticles in the alveolar hypophase is liable to cause increase in the production of collectins (SP-A, SP-D) to counter the xenobiotics at the cost of the secretion of phospholipid (PL) or other surface-active (SA) proteins (SP-B, SP-C). Increase in oxidative stress and lipid peroxidation leads to pulmonary inflammation, which can change the surfactant composition drastically and cause surfactant dysfunction [21]. Pulmonary inflammation is considered separately. Module I, as well as Module III, are shown schematically in Figure 2. The binding of phospholipids (PL) with NP is estimated using results from [22]. The process is modeled using Michaelis-Menten kinetics which depends on the surface area *A* of the particles and the available amount of surfactant at the alveolar interface. The depletion of PL is given by:
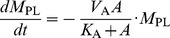
(12)where 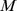 is the amount (in mol/ml) of PL, and 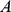 denotes the surface area of nanoparticles per ml of fluid (in m^2^/ml). The estimated Michaelis-Menten parameters for the NP-surfactant binding are summarized in Table 6. The loss in free PL due to binding to NPs also leads to loss in free surface area of the NPs. The loss in area and loss in PL are related as: 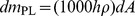, where 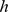 is the thickness of surfactant coating formed on the surface of particles (estimated to be 4 nm [20]), 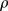 is the density of surfactant (estimated to be 1040 mg/ml by [23] = 1439.6 mol/ml), and the factor of 1000 is included to account for the conversion between ml/m^2^ and nm. 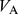 and 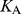 are the Michaelis-Menten parameters estimated using results from [22]. The nanoparticle balance equations in Module 1 calculate the number of NPs in different compartments, the number bound to PL, and the number which is free of PL.

**Table 6:** Binding properties of nanoparticles.

	*V*_A_ (mg/sec-ml)	*K*_A_(m^2^/ml)
Oxidized surface	5.1×10^−3^	1.03×10^−2^
Non-oxidized surface	3.581×10^−3^	1.131×10^−2^



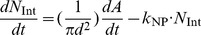
(13)

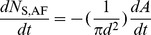
(14)

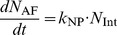
(15)



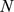
 denotes the number density of NPs (in number per ml) in various compartments and 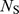 denotes the NPs coated with surfactant. The NPs coated with surfactant are considered to be transported into the alveolar fluid (AF) instantly because of the cyclical surfactant exchange during every breathing cycle, whereas a fraction of the naked NPs are transported to the AF, the fraction given by 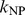.

### Cellular Uptake of Nanoparticles - Module III

NPs are taken up by alveolar cells via endocytosis or phagocytosis. This phenomenon plays a critical role in estimating exposure and fate of NPs in the biological system as the alveolar epithelial cells form the gateway to the circulatory system and hence to the entire body. Lai *et al.* [24] showed that charcoal NPs are significantly taken up by Type I cells, Type II cells, and macrophages. Cellular uptake of particles is influenced by particle type, size and surface charge [25]. The process of cellular uptake has been considered to be composed of two processes: delivery and adhesion of NPs onto the cell and uptake of NPs by the cell via endocytosis or phagocytosis. Adhesion of NPs onto cell surface is a function of particle size, surface zeta potential, and by the type of cell. Adhesion probability, 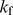 is modeled according to [25] as: 
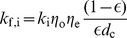
, where, 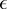 is the tissue porosity for the particular cell type, 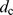 is the cell diameter, 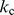 is a cell type dependent parameter, 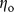,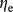, are the relative affinities of particle adhesion to the cell due to their size and surface potential respectively. Values of porosity and cell diameter for the alveolar cells are summarized in Table 7. 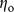 is a function of NP diameter 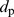 and the relation has been obtained for alveolar Mph from [26] and for other cells from [25]. 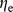 is a function of surface zeta potential of the NPs 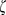 and the relation has been obtained for alveolar Mph from [27] and for AT1 and AT2 cells from [25] and [28]. Type I and Type II cells also differ intrinsically in their particle uptake properties due to different distribution of cationic and anionic binding sites on their surfaces [29]. Kemp *et al.* [30] compared particle uptake in Type I and Type II cells for both positively and negatively charged particles. The cell dependent parameter 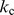 for AT1 and AT2 has been estimated using *in vitro* results from Kemp *et al.* [30]. Original NPs and NPs bound to surfactant would have different adhesion with cells. Ruge *et al.* [31] reported that the surface zeta potential of NPs after binding with PL and SP-A is −39.2 mV. Endocytosis and phagocytosis processes are modeled by Michaelis-Menten kinetics. Michaelis-Menten parameters for NP endocytosis by AT1 and AT2 were estimated from [32] where endocytosis of gold NPs by HeLa cells is reported. NP phagocytosis rate parameters were estimated from [33]. The uptake of NPs by cells is given by:
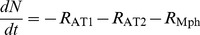
(16)

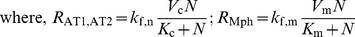
(17)

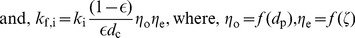
(18)


**Table 7:** Alveolar cell physical properties.

	AT1 cells	AT2 cells	Mph
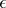 values*	0.04	0.97	0.99
Diameter, 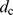 (m)	75**	10**	11.2†

* Values estimated from Clegg *et al.*, 2005 [56].

** From Chen *et al.*, 2004 [57].

† From Morgan & Talbot, 1988 [58].

### Elimination of Nanoparticles

Elimination of NPs from the alveolar region can involve 3 major routes [34]:

- Elimination of particles through the tracheobroncheal tree along with mucus into the gastro-intestinal system
- Elimination of particles by macrophages via phagocytosis or translocation into lymph nodes
- Diffusion of particles into blood circulation

Deposition of inhaled particles in the airway has been studied in multiple species. Raabe *et al.* [35] studied fractional deposition of ultrafine particles in mice. The results were extrapolated to get fractional deposition for 15 nm particles. Based on the observations from Raabe *et al.*, 40.26% of inhaled particles of 15 nm size can be predicted to reach the alveoli. The fractions deposited in the trachea, bronchi and alveoli were added to constitute the mass entering the pulmonary system during intratracheal (IT) instillation, which is 52.18% of the IT dose. In the absence of exact elimination fractions for each elimination pathway, the overall elimination estimated by Takenaka *et al.* [34] after IT dosing of 4–10 nm silver ultrafine particles in rats was used. Using the value of 52.18% of an IT dose reaching the alveoli, and the overall daily elimination estimated by Takenaka *et al.*, the elimination rate, 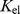, can be estimated to be 3.511×10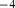 per min.

### Modeling Pulmonary Mechanics - Mechanics Module

**Figure 3:**
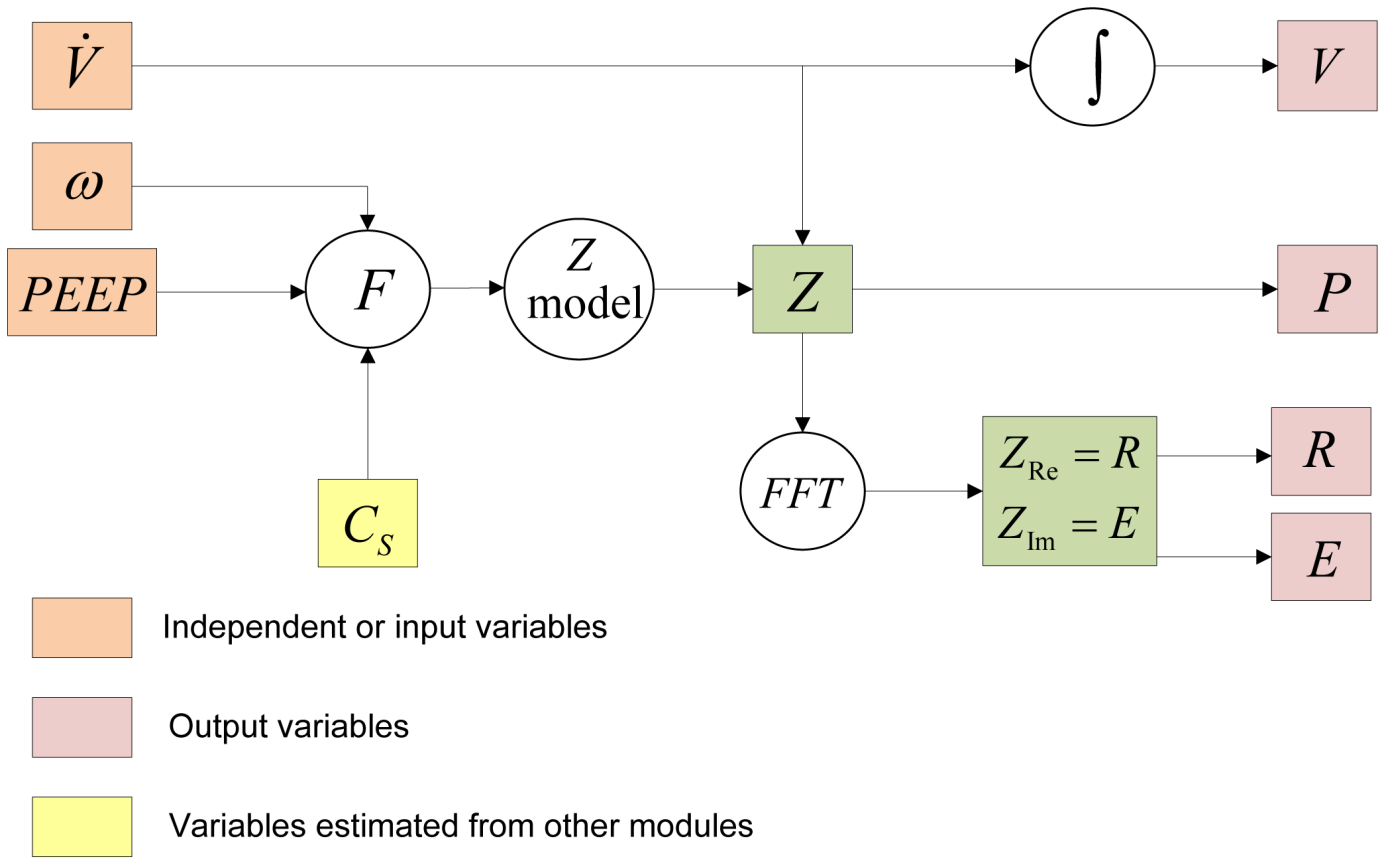
Schematic for modeling pulmonary mechanics in conjunction with surfactant concentration *C*_s_.

The mathematical model simulates the mechanical operation of the lung involving its cyclic expansion and compression and the dynamics occuring in the pulmonary hypophase (or BAL fluid), linking the two divergent phenomena by surfactant and its dynamic surface tension. The Mechanics Module is schematically shown in Figure 3.

#### Modeling pulmonary impedance

Pulmonary impedance can be described as the opposition to the flow of air into the lungs, and like electrical impedance, is defined as the ratio of the driving force causing the flow (in this case pressure, *P*) and the rate of flow (in this case air flow rate, 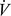). The relation between pressure and volume of air in lungs has been modeled since Otis *et al.*, 1956 [36], using the analogy of an electric R-C circuit, with pressure and flow rate analogous to voltage and electric current respectively. Hence, 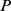 and 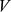 can be related as 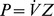, where 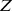 is the pulmonary impedance. Pulmonary impedance is intrinsically dependent on lung viscoelasticity. Various formulations have been developed over the years to relate various frequency dependent and independent lung parameters with impedance. Hildebrandt [37] first showed in 1970, by his experiments with cat lungs, that the viscoelastic modulus of this system varies linearly with the logarithm of time. Hantos and co-workers [38], [39] made modifications to Hildebrandt’s original theory with their Constant-Phase Model (CPM), where they decomposed the complex pulmonary impedance into components due to airway resistance (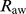), inertance (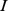), tissue-damping (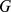), and tissue elastance (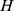).
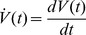
(19)

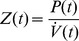
(20)

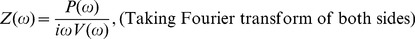
(21)


The mechanics equation which relates pulmonary air flow to pressure can be considered to have components due to inertial effects, airway resistance and the elastic forces in the lung. Accordingly the force balance for flow into and out of the lung can be written as a differential equation in time domain as:
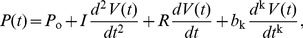
(22)where 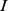 is the gas inertance responsible for inertial forces, 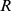 is the airway resistance, and 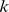 is a dimensionless number representing the dimensionality of the pulmonary elastic forces. Fourier transformation of each component in equation 22 helps transform the quantities from time (t) domain to frequency (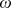) domain and makes the analysis more mathematically amenable by utilizing the theory of complex numbers. Fourier transformation of each term in equation 22, and adding them up produces real and imaginary parts of complex impedance in frequency domain, 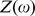, as:
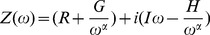
(23)where, 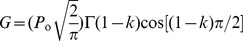, 
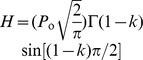
, and 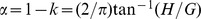. This is the form of the Constant Phase Model, the name arising out of the fact that the phase difference between the real and imaginary components of the equation is independent of frequency, 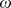. Physically, the real part of pulmonary impedance represents the physical impedance to airflow and energy loss due to impedance. The imaginary part of impedance represents energy storage due to the recoil forces in the lung. Following the analogy of an R-C circuit, the magnitude of impedance 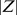 can be expressed as 
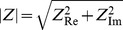
. The parameters R, G, and H are considered to be affected by surfactant concentrations. Change in surface concentrations transforms R, G, and H to R*, G*, and H*, where 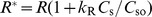, 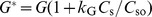 and 
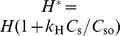
. The parameters 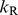, 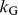, and 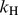 also depend on Positive End Expiratory Pressure (PEEP) and morphological changes in the lung and have been optimized based on lung function measurements in mice. The parameters 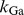, 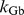, etc. capture the changes and injuries to the pulmonary system due to the NP administration.

### *In vivo* measurements

*In vivo* measurements of nanoparticle effects were done in experiments involving nine-week old C57-BL6 Jackson wild-type male mice. The animals (average body weight 24.82 g) were intra-tracheally dosed with 1 and 10 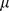
g/ml per g body weight of silver nanoparticles (nAg) and carbon black (CB) (Botelho *et al.,* Surface functionalization and diameter determine *in vivo* pulmonary response to silver nanospheres, *manuscript in preparation*) Intra-tracheal instillations and mechanical ventilation were conducted under ketamine/xylazine anesthesia, and all efforts were made to minimize suffering. Post-measurement, the animals were sacrificed using a lethal dose of ketamine/xylazine and exsanguination. Nanoparticle suspensions were prepared using 45 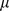
l of HBSS (Hank’s Balanced Salt Solution) and 5 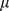
l of the large aggregate fraction from mouse BALF along with 0.05 and 0.5 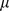
g per g body weight of NPs to make the final dose 1 and 10 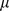
g/ml per g body weight respectively. Particles were mixed in solution using probe sonication immediately before intratracheal instillation. The dosed mice were rested for 1 day, 3 days and 7 days and then anesthetized and subjected to forced oscillatory breathing manoeuvres on a Flexivent (SCIREQ, Montreal, Canada) system for a spectrum of frequencies at different positive end expiratory pressures, which consists of measurements of overall pulmonary resistance (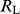) and elastance (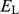) which are related to the real and imaginary parts of impedance as: 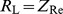 and 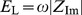. Additionally, post ventilation, the lungs of the mice were harvested and lavage fluid extracted for analyses. Total phospholipid was measured using the Bligh & Dyer method (1959), with extraction of the lipids with chloroform-methanol and subsequent measurement of total phosphate. Surfactant proteins B and D in mouse BALF were also estimated.

## Results

The model described above simulated the conditions of intratracheally exposed mice, and the subsequent forced oscillations. Simulations of the model were carried out for 10–20 nm citrate-stabilized silver NPs and carbon black (CB), with a surface zeta potential of −9.6 mV in BEGM (Basal Epithelial Growth Medium). The average size of the particles was calculated using the size distribution results obtained from particle characterization carried out by the manufacturer. The model was run for 10 additional days prior to dosing of NPs to let the levels of all surfactant components reach steady state reflecting the normal mouse physiological conditions. Consequently, the model was run for a total of 4, 6, and 10 days to simulate the 1, 3, and 7 day effects in the laboratory animals.

### Surfactant Components

**Figure 4:**
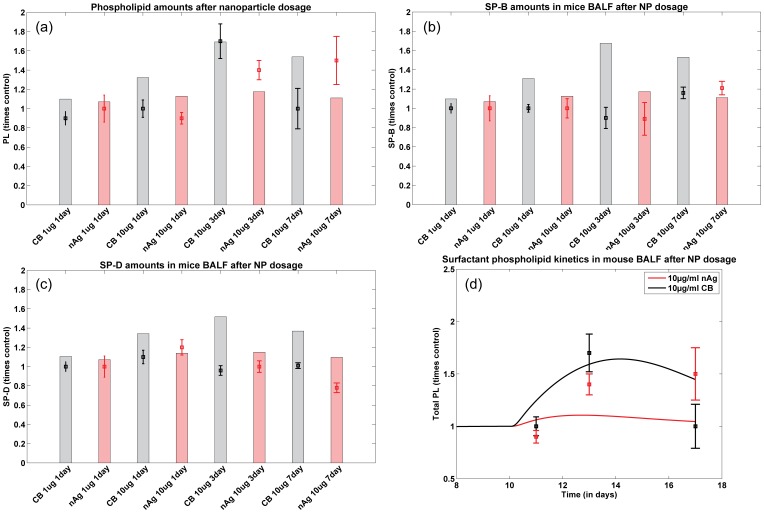
Comparison of predicted and measured values for different surfactant components (a) total PL, (b) SP-B, and, (c) SP-D in mice 1, 3, and 7 days after NP instillation; Bars represent model prediction for different NP intake scenarios and squares with error bars represent measurements in mice; (d) Comparison of PL kinetics in mice lavage fluid over 7 days (with 10 additional days prior to NP administration) with nAg shown in red and CB in black, lines representing model predictions and squares measurements in mice lung lavage.

Figure 4(a) shows a comparison of the model predictions and *in vivo* measurements of the total phospholipid (PL) levels for different treatment groups. Figure 4(d) shows the kinetics of total PL in mouse BALF over 7 days, for 10 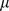
g/ml dose per g body weight of nAg and CB. The model predictions capture the kinetics in both cases in that it follows the increase around day 3 and the subsequent resolution in the PL levels in the alveolar fluid around day 7. The total PL includes free phosphoplipids in the BALF which are free for adsorption onto the alveolar surface as well as PL bound to NPs. Total PL increases after NP dosing, probably due to PL secretion from the Type II cells to make up for the loss in available free PL in the BALF causing an increase in free and bound PL in the BALF. The level of total PL subsequently decreases due to the elimination of the bound PL along with the NPs through cellular uptake. Figure 4(b,c) shows comparisons between *in vivo* mice lavage measurements and model predictions of surfactant proteins B and D respectively. The differences between the observed and predicted responses could be due to the effect of immune response on the surfactant secretion system especially on the collectins, which has not been considered in this version of the model.

### Cellular Uptake

**Figure 5:**
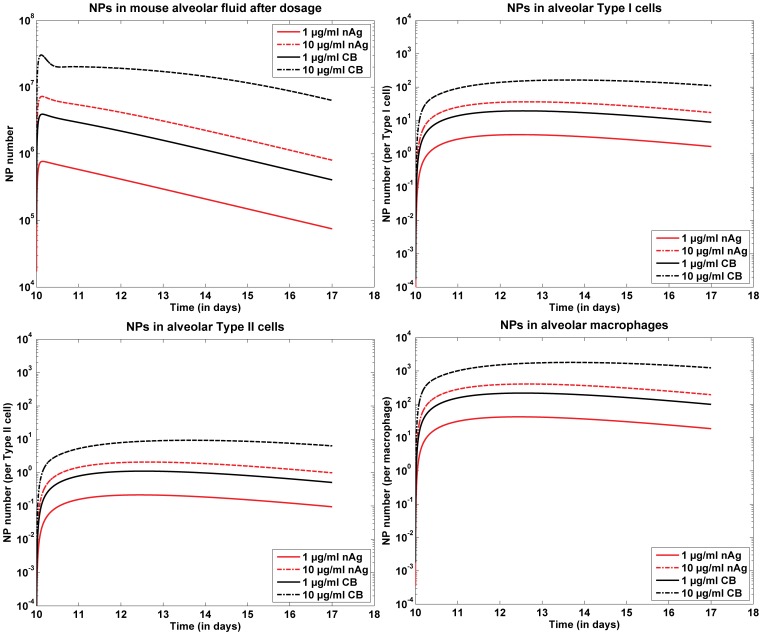
Model predictions for nAg kinetics in the alveolar region; (a) nAg kinetics in the alveolar fluid post dosing for 1 and 10
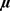
g/ml of dose per g body weight; (b) nAg uptake by various alveolar cells, representing total uptake by each type of cell for the 2 doses; (c) nAg uptake by various alveolar cells, representing uptake per cell by each type of cell.

Figure 5(a) shows the model results of the kinetics of nAg in the alveolar fluid over the entire time span of 10 days for both doses of nAg and CB. The smaller dose shows a much faster clearance through the alveolar region than the higher dose. Figures 5(b)-(d) show NP uptake by different cells in the alveolar region, alveolar type I cells, type II cells and alveolar macrophages. The results have been normalized by the number of cells in the alveolar population. Macrophages have a higher number of NPs per cell because macrophages are more efficient in the uptake of NPs than type I or type II cells. Type II cells have the least uptake of NPs. Both particles intrinsically have the same affinity for uptake by cells due to the same surface functionalization. The observed difference in uptake between nAg and CB is due to the fact that for the same mass dose of particles, CB have an order higher particle number than nAg.

### Lung Function

**Figure 6:**
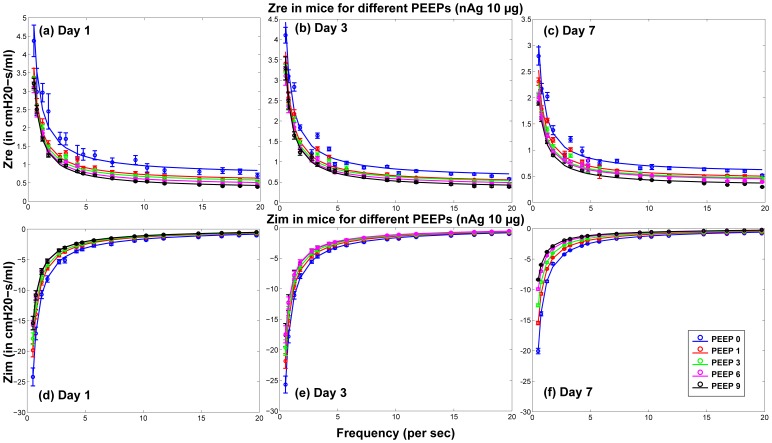
Comparison of predicted and measured values of real and imaginary part of pulmonary impedance in mice 1, 3, & 7 day post-dosing of 10 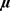
g of nAg (per g body weight) for different PEEPs (Circles and error bars represent measurements in mice and solid lines represent model results).

**Figure 7:**
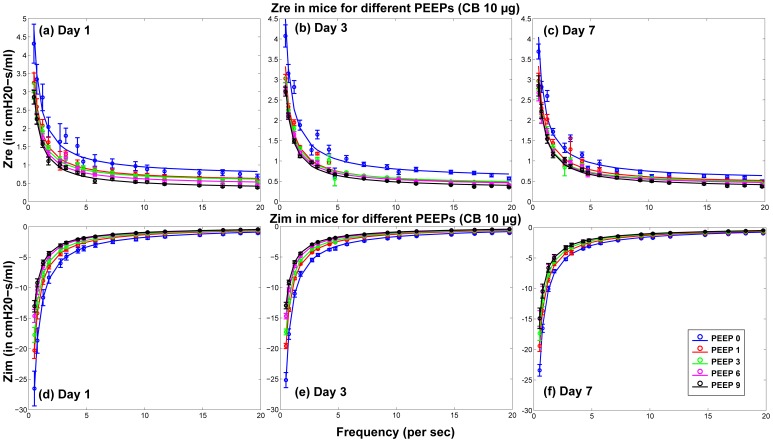
Comparison of predicted and measured values of real and imaginary part of pulmonary impedance in mice 1, 3, & 7 day post-dosing of 10 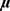
g of CB (per g body weight) for different PEEPs (Circles and error bars represent measurements in mice and solid lines represent model results).

**Figure 8:**
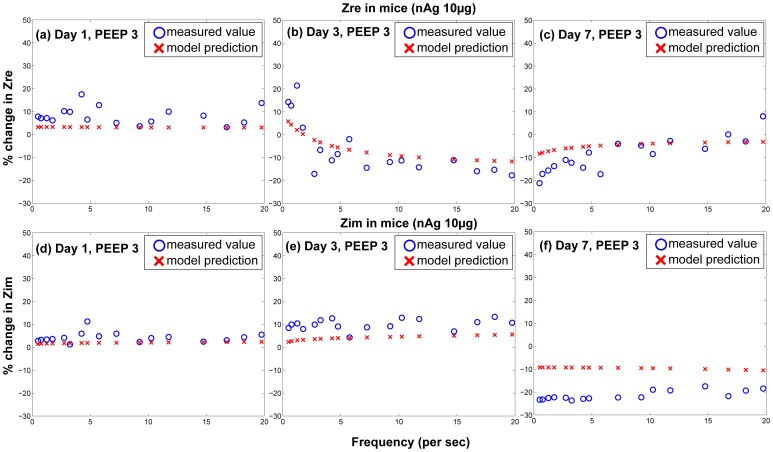
Comparison of differences between control and treated groups for model prediction and *in vivo* measurements of real and imaginary parts of pulmonary impedance in mice for a dose of 10 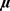
g of nAg per g body weight for PEEP = 3.

**Figure 9:**
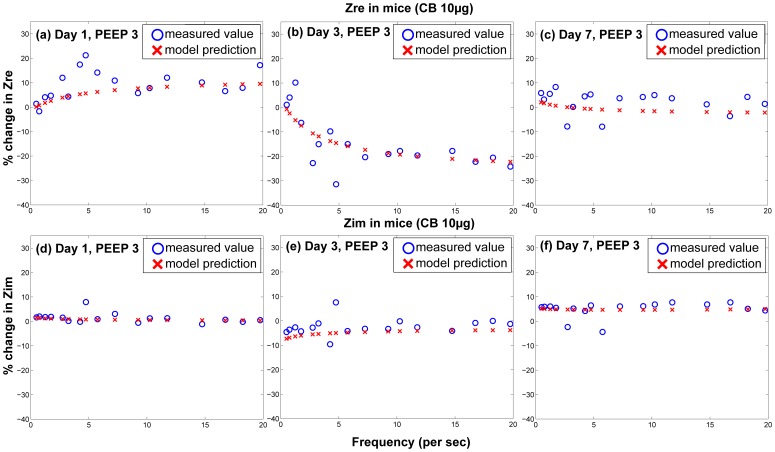
Comparison of differences between control and treated groups for model prediction and *in vivo* measurements of real and imaginary parts of pulmonary impedance in mice for a dose of 10 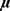
g of CB per g body weight for PEEP = 3.

**Figure 10:**
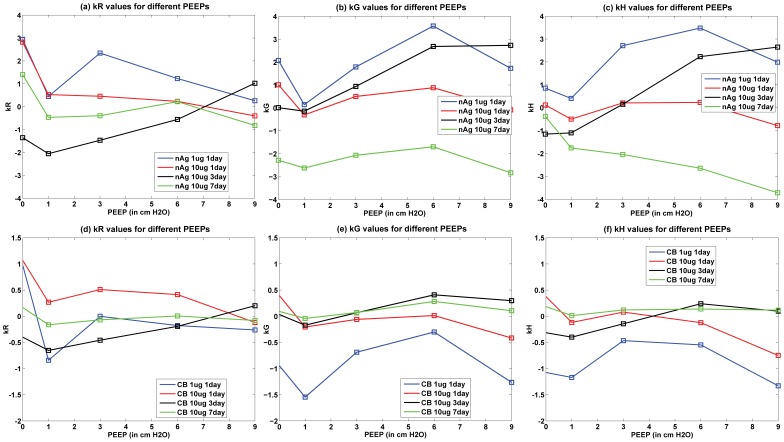
Modified Constant Phase Model linear parameters for surfactant dependent effects.

**Figure 11:**
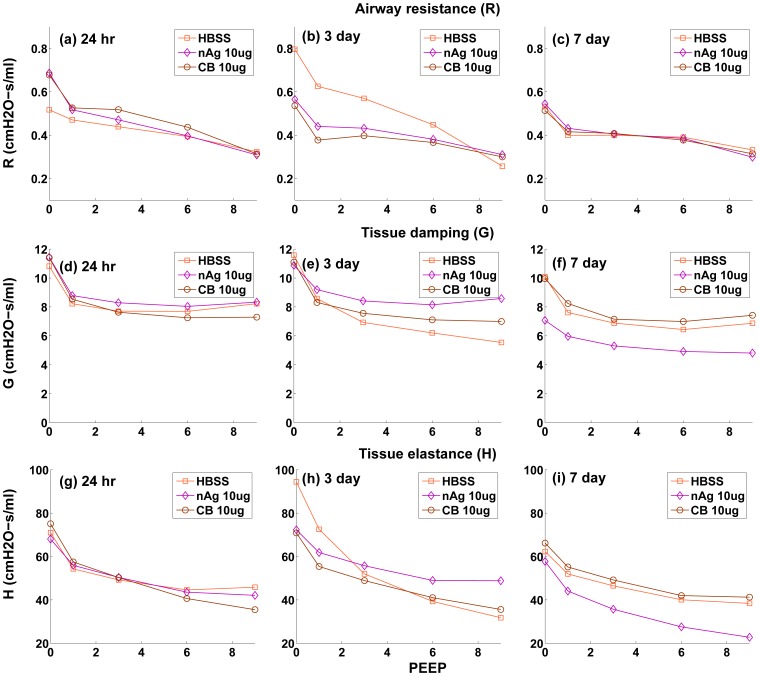
Variation of Constant Phase Model parameters R, G, & H with PEEP, at 1, 3, & 7 days post-dosing.

The surfactant amounts estimated by Module II of the model were used in the Mechanics Module to estimate resultant changes in alveolar surface tension and overall lung function. Lung function was estimated by computing overall lung resistance and elastance which correspond to the real and imaginary parts of lung impedance, which again is estimated as pressure/flow rate. Figure 6 shows comparisons between model results and measured values of 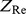 and 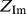 for 1, 3, and 7 days post-dosing of 10 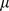
g nAg for different PEEPs. Figure 7 shows comparisons between model results and measured values of 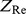 and 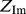 for 1, 3, and 7 days post-dosing of 10 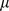
g CB for different PEEPs. Figures 8 and 9 show the difference of 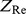 and 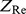 for treated mice and control mice, comparing *in vivo* measurements with model predictions, for PEEP = 3 for both types of NPs. The differences are calculated as: 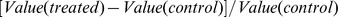. Only the values corresponding to PEEP = 3 are shown, but the parameters 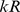, 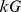, and 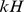 fitted to the data are shown for all values of PEEP in Figure 10. Figure 10 shows the values of the linear coefficients k, which were used to modify the traditional Constant Phase Model to account for the effects of changes in surfactant levels in the alveolar region. The values of the coefficients show a change from a PEEP of zero to a PEEP of 1, showing a change in the pulmonary response between the two conditions. Zero PEEP corresponds to a stressful condition for the lung and hence change in surfactant levels are expected to cause a much greater effect than at PEEPs of 1 or 3. There is also a difference in the response due to the two types of NPs. For CB, the values of the coefficients increased with increase in NP dose from 1 to 10 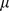
g, whereas for nAg the values decreased and an opposite effect is observed. nAg is an engineered nanoparticle, with a citrate-coating, whereas CB is used as a control particle. The difference in the surface coating can potentially elicit a different immune response from the cells. Overall tissue lung function can be influenced by a number of effects at the cell level including immune response and its subsequent effect on surfactant protein regulation. CB is generally known as one of the major causes for immune response in cells [40], [41], which is related to the regulation of surfactant proteins. Modeling the inflammatory response and its influence on the regulation of surfactant proteins is beyond the scope of the article and is in fact the subject of ongoing analysis by our team. The linear coefficients only capture the relationship between lung function and surfactant amount, so the opposite effect demonstrates that surfactant amount alone is not sufficient to explain the mechanism of observed changes in lung function. This suggests that surface-treatment not only affects surfactant binding and surfactant amount but also produces a different immune response in the cells. Figure 11 shows the change in the Constant Phase Model (CPM) parameters R, G, and H over different PEEPs and over days post-dosing.

## Discussion

The model successfully simulates the time dynamics of the pulmonary alveolar surfactant system in response to particle inhalation. Figure 4(b) shows the comparison of kinetics of total PL (both free and bound to NPs) in the alveolar region after dosing with nAg and CB.

The model relates surfactant PL levels in the alveolar region to changes in mechanical responses of the lung using a modified version of the Constant Phase Model (CPM). The differences in lung function values between treated and control mice (shown in Figures 8 & 9) follow similar trends as the total PL levels. The differences are minimal at 1 day, increase at 3 days and decrease again at 7 days. Among the Constant-Phase Model (CPM) parameters R, G, and H (Figure 10), R, which is related to upper airway resistance, is the least affected by NP dosage. The pattern of change in G and H seems to mimic the change in total surfactants, as there is appreciable change at 3 days which shows subsequent resolution at 7 days. The only exception is the appreciable lowering seen in G and H for nAg at 7 days which might be a result of prolonged injury and stress in the mice at 7 days post intra-tracheal instillation. This is also reflected in Figure 8(f), where there is an appreciable deviation between model and measurement at Day 7, which might be due to stress in the mouse pulmonary system over longer periods, due to the dosing mechanism. The intratracheal dosing mechanism, involving a tracheal cannula, produces some amount of injury in the mouse trachea which could worsen with time in some mice. As well, there might be some mouse-specific effects which may not be comparable to the control mice.

**Figure 12:**
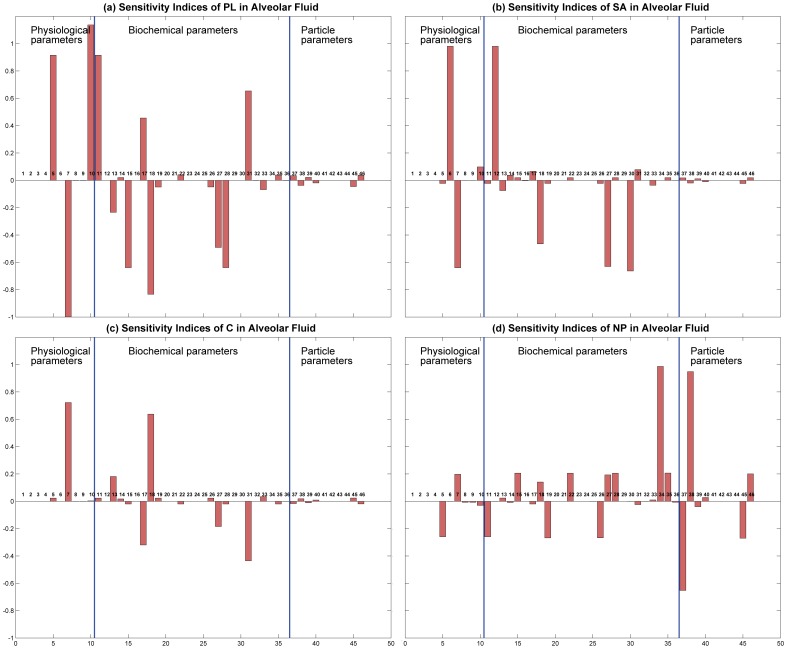
Sensitivity indices for 46 parameters in the toxicodynamic model; sensitivity has been calculated to different output variables (a) PL in alveolar fluid, (b) SA in alveolar fluid, (c) C in alveolar fluid, and (d) NPs in alveolar fluid.

Sensitivity analysis was carried out for the 46 parameters involved in the toxicodynamic model. The parameters have been grouped into physiological (10), biochemical (30), and particle specific parameters (6). Figure 12 shows the sensitivity indices as bar plots. The indices have been calculated for four output variables in the alveolar fluid: amount of PL, amount of SA, amount of C, and number of NPs. The indices have been calculated according to the formula:
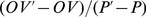
(24)where, 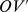 and 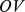 are the output variable values with changed parameter value, 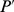, and with the original parameter value, 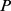, respectively. In Figure 12, the first 3 panels (a), (b), & (c) show negligible sensitivity in the 3rd group of parameters, which are particle specific, indicating that the surfactant secretion system is robust enough so that the total amount of PL, SA, or C in the alveolar fluid is not affected by particle properties. NP number in the alveolar region is affected by particle properties. Amount of PL is also the most sensitive variable and is affected the most by the parameter values.

This is the first attempt, to the authors’ knowledge, at mechanistically modeling the surfactant dynamics involving the pertinent cell types and processes like secretion, recycle, adsorption and degradation of surfactant phospholipid and proteins simultaneously. The model allows the regular biological dynamics to reach a steady state and mimics the introduction of xenobiotics to the alveolar micro-environment. The model considers important chemical properties of the NPs like size, material, and surface zeta potential, and properties of the cellular environment like cell diameter, fluid velocity in the vicinity, and cell packing density to model adhesion to cells which consequently affects cellular uptake. It has been demonstrated for citrate-stabilized NPs, but can be used to test other nanoparticles with different surface properties. The modular nature of the model allows individual testing of modules to wean out individual dynamics and can easily incorporate newer findings and additional *in vitro* chemical data regarding surfactant binding, cellular interactions and surfactant dynamics. Module III which relates to cellular uptake can be easily adapted to model other biological tissues with different cellular populations and cell properties. Using an extension of the Constant Phase Model, the multiscale model successfully quantifies the cellular-level dynamics in the alveolar micro-environment and relates them to organism-level changes. The model described here forms an important tool at assessing pulmonary effects due to a wide variety of nanoparticles. The model can be extended to other species and humans to assess pulmonary responses due to inhalation exposures to a variety of particulate matter of varying sizes and surface chemistries and forms an important step towards estimation of risk in a whole-body framework.
